# Kawasaki disease triggered by parvovirus infection: an atypical case report of two siblings

**DOI:** 10.1186/s13256-019-2028-5

**Published:** 2019-04-24

**Authors:** M. C. Maggio, R. Cimaz, A. Alaimo, C. Comparato, D. Di Lisi, G. Corsello

**Affiliations:** 1 University Department promise “G. D’Alessandro”, University of Palermo, Palermo, Italy; 2 NEUROFARBA Department, University of Florence, Florence, Italy; 3 Azienda Ospedaliero Universitaria (AOU) Meyer, Florence, Italy; 4 Paediatric Cardiology Unit, Children Hospital “G. Di Cristina”, ARNAS, Palermo, Italy

**Keywords:** Kawasaki disease, Anakinra, Kawasaki shock syndrome

## Abstract

**Background:**

There are reports of the familial occurrence of Kawasaki disease but only a few reports described Kawasaki disease in siblings. However, the familial cases were not simultaneous. In these patients the idea of infective agents as trigger must be considered.

**Case presentation:**

We describe two siblings with atypical presentations of Kawasaki disease; the sister was first diagnosed as having parvovirus infection with anemia and the brother was diagnosed as having myocarditis.

The first patient was a 9-month-old Caucasian girl with fever, conjunctivitis, rash, and pharyngitis, and later she had cervical adenopathy, diarrhea and vomiting, leukocytosis, and anemia, which were explained by positive immunoglobulin M against parvovirus. However, coronary artery lesions with aneurysms were documented at day 26 after fever onset. An infusion of intravenous immunoglobulin and high doses of steroids were not efficacious to resolve the coronary lesions. She was treated with anakinra, despite a laboratory test not showing inflammation, with prompt and progressive improvement of coronary lesions.

Her 7-year-old Caucasian brother presented vomiting and fever at the same time as she was unwell, which spontaneously resolved after 4 days. Four days later, he again presented with fever with abdominal pain, associated with tachypnea, stasis at the pulmonary bases, tachycardia, gallop rhythm, hypotension, secondary anuria, and hepatomegaly. An echocardiogram revealed a severe hypokinesia, with a severe reduction of the ejection fraction (20%).

He had an increase of immunoglobulin M anti-parvovirus, tested for the index case of his sister, confirming the suspicion of viral myocarditis. He received dopamine, dobutamine, furosemide plus steroids, with a progressive increase of the ejection fraction to 50%. However, evaluating his sister’s history, the brother showed a myocardial dysfunction secondary to Kawasaki shock syndrome.

**Conclusions:**

We report on familial Kawasaki disease in two siblings which had the same infectious trigger (a documented parvovirus infection). The brother was diagnosed as having post-viral myocarditis. However, in view of the two different and simultaneous evolutions, the girl showed Kawasaki disease with late coronary artery lesions and aneurysms, whereas the brother showed Kawasaki shock syndrome with myocardial dysfunction.

We stress the effectiveness of anakinra in non-responder Kawasaki disease and the efficacy on coronary aneurysms.

## Background

There are reports of the familial occurrence of Kawasaki disease (KD) but only a few reports described KD in siblings [1]. However, the familial cases were reported at different times, not simultaneously. Epidemiological studies indicated that specific viral and/or bacterial infections and an underlying genetics may trigger KD. Furthermore, the detection of a specific infectious agent, which could explain the clinical course of atypical or incomplete KD, may delay the correct diagnosis, with an increased risk of coronary artery lesions (CAL).

Treatment with intravenous immunoglobulin (IVIG) is useful if started within 10 days from the beginning of fever. When a delay is due to incomplete and/or atypical KD, an increased risk of CAL is reported [2].

In non-responders, with a severe outcome and/or high risk of CAL, a more aggressive treatment must be considered, with high doses of steroids, anti-interleukin (IL)-1 biologic drugs, and immune suppressive drugs [3].

Anakinra, a recombinant antagonist of IL-1 receptor, inhibits binding of IL-1 alpha and beta with the receptor and, in this way, down-regulates IL-1-mediated inflammation. KD, considered an autoinflammatory disease, recognizes in IL-1 the therapeutic target to blockade systemic symptoms and CAL [4].

We report the clinical cases of a brother and sister who were admitted to two different units of our Children Hospital with a late diagnosis of severe KD, in whom parvovirus infection seemed to be the probable triggering factor.

## Case presentation

### Case number 1

A 9-month-old Caucasian girl presented to our pediatric unit with fever, pallor, bilateral non-secreting conjunctivitis, and rash. Anamnestic records revealed that 12 days before she had remittent fever, which spontaneously resolved in 5 days. Fever started again after 3 days, associated with pharyngitis, and, later, with cervical adenopathy, diarrhea, and vomiting. She was treated with amoxicillin plus clavulanic acid and steroids, without defervescence. At admission, 9 days after fever onset, she showed fever, conjunctivitis, pharyngitis, generalized rash, and bilateral cervical adenopathy. Hematological parameters revealed: leukocytes, 18,000/mm^3^ with neutrophils of 8520/mm^3^, lymphocytes of 6250/mm^3^, and monocytes of 1930/mm^3^; hemoglobin, 9.1 g/dl; platelets, 318,000/mm^3^; and transaminases, albumin, natremia, and urine analysis in the normal range. Her C-reactive protein (CRP) was 2.31 mg/dl; her erythrocyte sedimentation rate (ESR) was 120.

An electrocardiogram (ECG) and echocardiography were normal, including coronary *Z*-scores. IgM and IgG against Epstein–Barr virus, cytomegalovirus, and parvovirus were tested, and she showed positive IgM against parvovirus. This was confirmed at further testing after 10 days. She was treated with clarithromycin and obtained quick defervescence. A diagnosis of parvovirus infection with severe anemia was made. For this reason and because of the prompt defervescence, it was exclusively treated as a viral infection. During follow-up, further cardiologic evaluation was done because of the risk of pericarditis secondary to the parvovirus infection, and at day 26 after fever onset, CAL were documented, with: a proximal right coronary artery *Z*-score of 6.02; left main coronary *Z*-score of 5.72; and left anterior descending *Z*-score of 5.78. Coronary artery *Z*-scores are commonly used for decisions in KD management and decisions on treatment, even if *Z*-scores show variations based on the *Z*-scores formula used for larger coronary artery dimensions [5]. However, the *Z*-score value for CAL is useful for the follow-up of a patient and for testing the response to treatment.

The child was promptly treated with IVIG at the dosage of 2 g/kg plus acetylsalicylic acid (ASA) at the dosage of 5 mg/kg per day. Despite treatment, a further echocardiographic evaluation showed worsening of CAL: proximal right coronary artery *Z*-score of 5.93; left main coronary *Z*-score of 5.63; and left anterior descending *Z*-score of 5.39, which showed a saccular aneurysm of 2.9 mm of diameter (*Z*-score of 5.08).

A laboratory test did not show inflammation, but the girl was treated with three bolus doses of intravenously administered methylprednisolone at 30 mg/kg per dose. The *Z*-score of CAL did not change. Informed consent by parents was obtained, and our patient was treated with anakinra at the dosage of 4 mg/kg per day. She showed a progressive improvement of CAL and after 25 days of anti-IL-1 treatment, her proximal right coronary artery *Z*-score was 0.93, left main coronary *Z*-score 4.02, and left anterior descending *Z*-score 2.93. Treatment was continued for 2 months, at which point *Z*-scores normalized (see Table 1).

**Table 1:** Coronary artery lesions (*Z*-scores) substantial improvement during anakinra treatment

Days of echocardiography	Proximal right coronary artery mm (normal values 0.82–2.20)	Proximal right coronary artery *Z*-score	Left main coronary mm (normal values 1.06–2.42)	Left main coronary *Z*-score	Left anterior descending mm (normal values 0.7–1.97)	Left anterior descending *Z*-score
Before IVIG	3.5	5.93	3.6	5.63	3.1	5.40
Before anti-IL-1	3.5	5.93	3.6	5.63	3	5.39
T = 4 days after anti-IL-1	3.5	5.93	3.6	5.63	3	5.39
T = 25 days after anti-IL-1	1.8	0.93	3.05	4.02	2.22	2.93
T = 40 days after anti-IL-1	1.8	0.93	2.9	3.46	2.2	2.77
T = 71 days after anti-IL-1	1.6	0.27	2.5	2.19	1.8	1.43

*IL* interleukin, *IVIG* intravenous immunoglobulin

The first diagnosis in this patient was a parvovirus-related infection, which explained the clinical manifestations and the clinical course of the disease. The diagnosis of KD was reached late; a control echocardiogram showed CAL. Mild CAL are described in systemic juvenile arthritis, in febrile diseases, and in infectious diseases such as Mediterranean spotted fever [6]. Aneurysms, conversely, are typical of KD.

IVIG did not arrest the worsening of CAL, which were stabilized after three doses of methylprednisolone. However, our patient received IVIG 26 days after the fever started and this delay can explain the poor response. In fact, a delay in IVIG infusion is recognized as a risk factor for non-response to first-line treatment, as demonstrated in our population [6, 7].

### Case number 2

A 7-year-old Caucasian boy, the brother of Case number 1, presented fever (38 °C) and vomiting at the same time as his sister, which spontaneously resolved after 4 days. Four days later, he again had fever, abdominal pain, tachycardia, and tachypnea. He was admitted to our cardiologic unit. He showed pallor, tachypnea, stasis at the pulmonary bases, tachycardia (180 beats/minute), gallop rhythm, hypotension, and secondary anuria hepatomegaly with pain at palpation. Hematological tests evidenced: leukocytes of 24,680/mm^3^ with neutrophils of 19,744/mm^3^, lymphocytes of 3430/mm^3^, and monocytes of 960/mm^3^; hemoglobin, 10.4 g/dl; platelets, 632,000/mm^3^; CRP, 0.24 mg/dl; aspartate aminotransferase (AST), alanine aminotransferase (ALT), and gamma-glutamyltransferase (gamma-GT) in the normal range; creatine phosphokinase (CPK), 773 mg/dl; creatinine, 0.77 mg/dl; and blood urea nitrogen (BUN), 111 mg/dl. He had elevated myocardial necrotic enzymes (c-troponin T, 91.4 ng/l) and pro-brain natriuretic peptide (BNP) > 70,000.

An echocardiogram revealed that the left ventricle had a normal diameter (telediastolic diameter, 40 mm; *Z*-score, 0.37) and generalized hypokinesia, with a severe reduction of the ejection fraction (EF) (20–25%); the left atrium was dilated (diameter, 35 mm; *Z*-score, 3.3) and the mitral valve had a moderate insufficiency. The right ventricle had normal dimension; the tricuspid valve showed a moderate insufficiency. His suprahepatic veins were dilated. No pulmonary hypertension was documented. He received dopamine (5 gamma/kg per minute), dobutamine (7 gamma/kg per minute), furosemide (1 mg/kg) plus steroids (2 mg/kg). Clinical signs, echocardiographic parameters, and plasmatic enzymes showed a progressive but slow improvement. Sixteen days later, his EF was 45%; however, a persistent septal hypokinesia was documented. He continued to receive treatment with furosemide and enalapril. Specific serological tests were performed to exclude Epstein–Barr virus (for the skin rash, associated with fever and hepatic cholangitis) [8] and coxsackie virus infection (for fever and myocarditis) [9]. A nasal swab for influenza and parainfluenza virus was negative. However, the index case of the sister suggested we should run a serology test for anti-parvovirus, and we found increased IgM anti-parvovirus with low IgG.

A cardiologic follow-up revealed: a further EF improvement (50%); left ventricle was 38 mm (normal value: 32.7–45.5); *Z*-score, 0.15. His right atrium and ventricle were in the normal range for diameters and kinesis.

## Discussion and conclusions

In the international literature cases of siblings simultaneously affected by KD are rare; however, familial recurrence of KD is documented [1]. In these cases, a genetic background can be suspected. However, simultaneous manifestations of the disease induce research for an infective trigger, as documented in our patients. Parvovirus can explain fever, rash, and anemia; these symptoms were detected in our children in the first phase of their illness, which was considered an infective disease. Furthermore, pericarditis and/or myocarditis are possible cardiac complications in parvovirus infection. For this reason, Case number 2 was admitted to our cardiologic unit and treated as a patient with parvovirus-induced myocarditis.

CAL are not imputable to parvovirus infection but to KD and the delay in the diagnosis in Case number 1 was associated with the worsening of the vasculitis even though our patient was treated with IVIG. After high doses of steroids, no worsening of CAL was documented by echography; however, the off-label treatment with anakinra had a crucial role in reversing aneurysms.

In conclusion, we report on familial KD in two siblings which had the same infectious trigger (parvovirus). The brother was diagnosed as having post-viral myocarditis. However, in view of the two different and simultaneous evolutions, the girl showed KD with late CAL and aneurysms, whereas the brother had Kawasaki shock syndrome with myocardial dysfunction. Viral illnesses are well-documented triggers of KD; however, in these cases the rarity is in the simultaneous occurrence in two siblings, albeit with different phenotypes. Furthermore, both the children showed a severe cardiac involvement, which, in part, could be explained by the parvovirus tropism specific for myocardial tissue. Of note, the girl had aneurysms which almost disappeared after anakinra treatment, a therapy which has been recently shown to be promising for this disease. The significant response to anakinra (an off-label treatment in this condition) is in fact supported by other reports [4, 10–12]; by contrast, anti-tumor necrosis factor (TNF)-alfa drugs are effective especially in the control of fever and inflammatory parameters [13].

In addition, the follow-up of KD needs to consider a late evolution in CAL, especially in patients with atypical KD; this circumstance may be a cause of misdiagnosis.

The brother was diagnosed and treated as having myocarditis secondary to parvovirus infection. It is well known, in fact, that parvovirus can elicit myocardial dysfunction with reduced EF, secondary to myocarditis. However, the case of the sister highlighted that the possible correct diagnosis in the boy was Kawasaki shock syndrome induced by a viral trigger [14].

## Abbreviations

ALT: Alanine aminotransferase
ASA: Acetylsalicylic acid
AST: Aspartate aminotransferase
BNP: Pro-brain natriuretic peptide
BUN: Blood urea nitrogen
CAL: Coronary artery lesions
CPK: Creatine phosphokinase
CRP: C-reactive protein
ECG: Electrocardiogram
EF: Ejection fraction
ESR: Erythrocyte sedimentation rate
gamma-GT: Gamma-glutamyltransferase
IL: Interleukin
IVIG: Intravenous immunoglobulin
KD: Kawasaki disease
TNF: Tumor necrosis factor
